# Genetic Adaptations of the Tibetan Pig to High-Altitude Hypoxia on the Qinghai–Tibet Plateau

**DOI:** 10.3390/ijms252011303

**Published:** 2024-10-21

**Authors:** Yanan Yang, Haonan Yuan, Boyuan Yao, Shengguo Zhao, Xinrong Wang, Linna Xu, Lingyun Zhang

**Affiliations:** College of Animal Science and Technology, Gansu Agricultural University, Lanzhou 730070, China; hnyuan1101@163.com (H.Y.); 15030068709@163.com (B.Y.); zhaosg@gsau.edu.cn (S.Z.); wangxr@gsau.edu.cn (X.W.); xulinna126@126.com (L.X.); zhanglingyun1110@163.com (L.Z.)

**Keywords:** Tibetan pigs, nature selection, high-altitude adaptation, whole-genome sequencing

## Abstract

The Tibetan Plateau’s distinctive high-altitude environment, marked by extreme cold and reduced oxygen levels, presents considerable survival challenges for both humans and mammals. Natural selection has led to the accumulation of adaptive mutations in Tibetan pigs, enabling them to develop distinctive adaptive phenotypes. Here, we aim to uncover the genetic mechanisms underlying the adaptation of Tibetan pigs to high-altitude hypoxia. Therefore, we conducted a systematic analysis of 140 whole-genome sequencing (WGS) data points from different representing pig populations. Our analysis identified a total of 27,614,561 mutations, including 22,386,319 single-nucleotide variants (SNVs) and 5,228,242 insertions/deletions (INDELs, size < 50 bp). A total of 11% (2,678,569) of the SNVs were newly identified in our project, significantly expanding the dataset of genetic variants in Tibetan pigs. Compared to other pig breeds, Tibetan pigs are uniquely adapted to high-altitude environments, exhibiting the highest genetic diversity and the lowest inbreeding coefficient. Employing the composite of multiple signals (CMS) method, we scanned the genome-wide Darwinian positive selection signals and identified 32,499 Tibetan pig positively selected SNVs (TBPSSs) and 129 selected genes (TBPSGs), including 213 newly discovered genes. Notably, we identified eight genes (*PHACTR1*, *SFI1*, *EPM2A*, *SLC30A7*, *NKAIN2*, *TNNI3K*, and *PLIN2*) with strong nature selection signals. They are likely to improve cardiorespiratory function and fat metabolism to help Tibetan pigs become adapted to the high-altitude environment. These findings provide new insights into the genetic mechanisms of high-altitude adaptation and the adaptive phenotypes of Tibetan pigs.

## 1. Introduction

The extreme cold and hypoxic conditions of the Qinghai–Tibet Plateau present significant challenges for the survival and development of humans and mammals. Through long-term natural selection, plateau-specific animals such as the Tibetan pig (*Sus scrofa*) [1,2], the Tibetan sheep (*Ovis aries*) [3], yak (*Bos grunnien*) [4,5], the Tibetan chicken [6], and plateau pika [7], along with humans (Tibetans) [8], have collectively formed a “group advantage” in adaptive evolution research, providing a luxuriant genetic resource for unraveling the genetic mechanisms underlying adaptation to hypoxia on the plateau.

As the unique pig breed adapted to the plateau, the Tibetan pig has exhibited significant physiological and genetic adaptations [2,9]. Research has shown that the Tibetan pig copes with the intense ultraviolet radiation (UVR) of the plateau through black skin and longer, tougher bristles [10], and it enhances its cardiopulmonary system’s oxygen-carrying capacity through thicker alveolar septa, developed capillaries, and higher hemoglobin levels to adapt to the plateau environment [2,9,11].

Omics studies, including genomics and transcriptomics, have revealed that the adaptation of the Tibetan pig to the plateau is a complex process involving systemic organ adaptations. Research reveals that gene family expansion events are less frequent in Tibetan pigs compared to Duroc pigs. Notably, the rapidly evolving genes in Tibetan pigs are associated with fat deposition, thermogenesis, and the development of cardiac and skeletal muscles. These adaptations likely reflect the necessity to enhance biomass production efficiency in resource-limited, high-altitude environments [12,13,14]. Transcriptome studies have revealed that the differentially expressed genes (DEGs) in Tibetan pigs and migration pigs are significantly enriched in pathways related to vascular morphogenesis, vascular development, and cardiac development, which are associated with hypoxic responses and the cardiovascular system, suggesting that superior cardiopulmonary function may be a key feature of the Tibetan pig’s adaptation to the plateau [15,16]. Whole-genome positive selection signal analysis showed that multiple genes are involved in the Tibetan pig’s cardiovascular system, lung and gas exchange, energy metabolism, and immunity and apoptosis [1,17]. Notably, mutations of *TMPRSS6*, *NFE2*, and *THSD7A* in the Tibetan pig regulate red blood cell maturation and differentiation and embryonic angiogenesis, optimizing oxygen transport and increasing blood flow to the uteroplacental circulation in hypoxic conditions, ultimately maintaining a good blood oxygen degree of saturation and reproductive fitness [11,18].

In summary, the Tibetan pig has become well adapted to the plateau and exhibits unique adaptive traits likely underpinned by corresponding genetic foundations. Over the past few decades, multiple studies have reported 209 genes associated with high-altitude adaptation in Tibetan pigs [9,11,19,20]. However, due to limited sample sizes, bias of statistical methods, and the lack of control groups, these genes have not been consistently replicated across multiple studies, significantly limiting the elucidation of adaptive phenotypes and the utilization of germplasm resources in the Tibetan pig. Here, we systematically analyzed 140 whole-genome sequencing (WGS) data points from different pig breeds, identified genome-wide Darwinian positive selection signals in the Tibetan pig, and provided a robust set of nature-selected genes, laying the genetic foundation for the analysis of adaptive phenotypes, plateau adaptation research, and the development of germplasm resources in the Tibetan pig.

## 2. Result

### 2.1. Genomic Dataset and Population Structure

**Figure 1:**
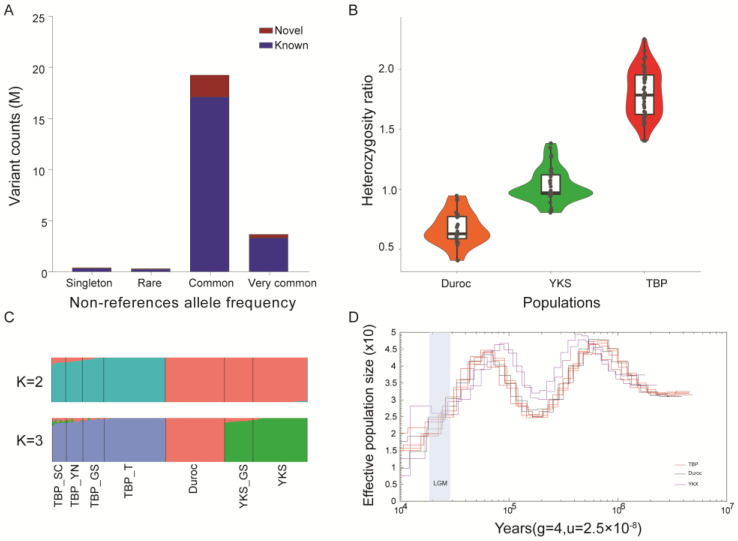
Genomic variants across the entire pig genome and population structure. (**A**) Analysis of non-reference allele frequencies across the entire genome of Tibetan pigs identified as known and novel variants using dbSNP_138 as a reference. We discovered that 11% (2,678,569) of these SNVs are novel. (**B**) The distribution of heterozygosity ratios among different pig breeds showed that Tibetan pigs have the highest heterozygosity ratio (*p* = 7.07 × 10^−34^, *p* = 1.48 × 10^−37^), with significant differences determined by *t*-test. (**C**) Population structure analysis revealed that Tibetan pigs (TBPs) are a relatively distinct highland breed in China. Although gene flow from foreign breeds was detected in 0.1–3% of Tibetan pigs from Sichuan, Yunnan, and Gansu, this component likely represents shared ancestry or gene exchange; YKS, Yorkshire; TBP, Tibetan pig; SC, Sichuan; GS, Gansu; T, Tibetan; YN, Yunnan. (**D**) Population history analysis indicated that during the Last Glacial Maximum, both TBPs and other global pig populations experienced reductions in population size, consistent with effective population changes across species during this period. Post-glacial domestication events likely led to further declines in the effective population size of YKS and DU pigs.

We performed whole-genome resequencing (WGS) on 30 pigs from different breeds (10 Tibetan pigs, 10 Yorkshire pigs, and 10 Duroc pigs) and integrated our data with 110 previously published WGS datasets, forming a comprehensive dataset for this study [1,2,9,11,19,20,21] (Supplementary Table S1). Using the well-trained GATK4(v.4.6.0) pipeline, we successfully identified 27,614,561 mutations, including 22,386,319 single-nucleotide variants (SNVs) and 5,228,242 insertions and deletions (INDELs, size < 50 bp). A detailed analysis of the non-reference allele frequencies (NRFs) revealed that 11% (2,678,569) of these SNVs are novel (do not exist in dbSNP_138, Figure 1A). Among these novel SNVs, 7% (1,666,224) are classified as common variants with a minor allele frequency (MAF > 0.1) (Figure 1A). Principal component analysis (PCA) revealed a clear separation between Tibetan pigs and other plain pigs along PC1, confirming the robustness and representativeness of our dataset (Supplementary Figure S1).

Genetic diversity is crucial for the evaluation, development, and conservation of germplasm resources. We assessed the genetic diversity of Tibetan pigs using heterozygosity ratios, inbreeding coefficients (IBCs), and the number of variants carried by individuals. The results indicated that Tibetan pigs had higher heterozygosity ratios (TBP had a higher heterozygosity ratio, *p* = 7.07 × 10^−34^, *p* = 1.48 × 10^−37^) (Figure 1B) and lower IBCs compared to Yorkshire and Duroc pigs (Supplementary Figure S2). These findings were reflected in the number of variant sites and linkage disequilibrium (LD) decay (Supplementary Figure S2). LD decay indicates genetic structure and is associated with genetic diversity. Tibetan pigs showed the fastest LD decay, while Yorkshire and Duroc pigs had the slowest, suggesting higher genetic diversity in Tibetan pigs, consistent with their grazing lifestyle.

The TBP population relationship illustrated by the ADMIXTURE analysis was in accordance with the pattern revealed by PCA (Figure 1C). Tibetan pigs were identified as a relatively distinct highland breed in China. Despite detecting 0.1–3% gene flow from foreign breeds in Tibetan pigs from Sichuan, Yunnan, and Gansu, it is well known that Duroc pigs incorporated Chinese pig genes during their development. Moreover, recent breeding of migration pigs in low-altitude areas around the Tibetan Plateau has facilitated gene flow. Therefore, the 3% foreign component in Tibetan pigs likely represents shared ancestry or gene flow from migration pigs.

To reconstruct historical population dynamics, we used the pairwise sequential MSMC2 and PSMC methods to estimate the effective population size (*N*e) of ancestral populations. Our results indicated a significant and prolonged bottleneck in all pig breeds from over 100,000 to 130,000 years ago. During the Last Glacial Maximum, all pig populations were affected. In the post-glacial period, the effective population size of many plains pig breeds decreased due to intensified artificial breeding driven by agricultural expansion, while the Tibetan pig population remained relatively stable (Figure 1D).

### 2.2. Genome-Wide Scan for Darwin Positive Selection Signatures in TBP

**Figure 2:**
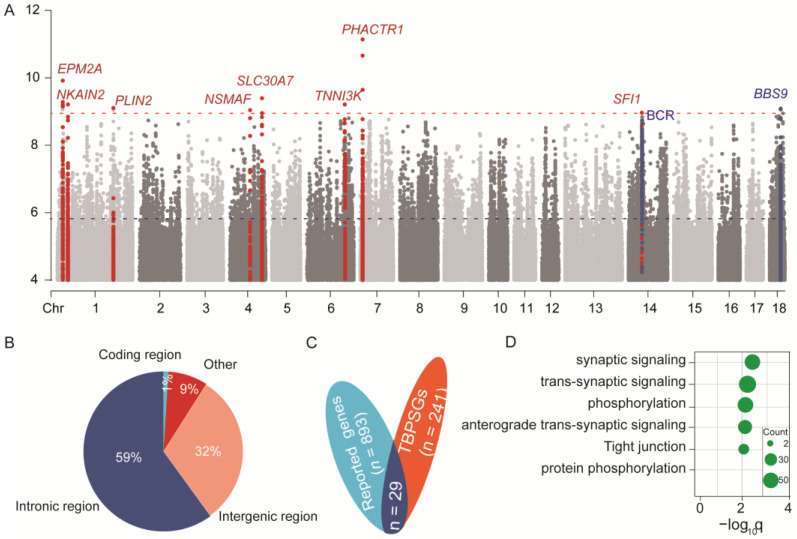
Genome-wide scan for genetic positive selection signatures in TBPs. (**A**) The distributions of TBPSGs in the TBP genome. The top 10 TCSGs at the genome-wide significant loci are highlighted; the red and blue lines represent the top 10 gene thresholds and the top 5 0.5% thresholds. (**B**) Annotations of the 32,499 SNVs with a CMS score > 0.5% (5.67) by VEP. (**C**) A Venn diagram of the TBPSGs, published genes, and the report genes from multiple studies (see Supplementary Table S8). (**D**) KEGG and GO enrichment analysis by g: Profiler. The gene counts are shown as the size of the circle.

Long-term natural selection has enabled Tibetan pigs (TBPs) to become well adapted to high-altitude environments. The composite of multiple signals (CMS) method is employed to detect genome-wide Darwinian positive selection signals in TBPs [22] (see the Methods Section). The top 0.5% of CMS scores for genome-wide SNVs were selected as TBP-selected SNVs (TBPSSs); as a result, we ended up with a set of 32,499 TBPSSs (Supplementary Table S2, Figure 2A) which are located in 270 independent genomic regions, with 29 regions not overlapping any genes and represented by 241 peak genes, referred to as the TBP selection genes (TBPSGs). Of the 241 TSNGs, 28 were reported in previous studies, and the other 213 were newly identified genes (Figure 2B,C, Supplementary Table S3).

Functional annotation of 32,499 TBPSSs revealed that most loci were located in intronic regions (59%), 28% in intergenic regions, and only 1% (*n* = 119) in coding regions, which included 31 missense mutations, 91 synonymous mutations, and 1 start-loss mutation (Figure 2B, Supplementary Tables S2 and S4). Compared to migration pigs, rs321821024 (*FBXO30*) and rs81217606 (*BPNT1*) were significantly enriched in TBPs (>68.67%) (Supplementary Table S4). Homozygous or heterozygous knockout mice for *FBXO30* and *BPNT1* exhibit phenotypes of metabolic disorders, skeletal abnormalities, and reduced fitness (see MGI, www.informatics.jax.org, accessed on 6 October 2024), indicating that these genes may play roles in TBP fitness and metabolic regulation.

For 241 TBPSGs, KEGG and GO term enrichment analysis showed significant enrichment in synaptic signaling and phosphorylation processes (Figure 2D). Phosphorylation is crucial for maintaining life processes, suggesting that TBPs may harbor a group of genes related to phosphorylation to help them maintain normal energy metabolism under hypoxic conditions. Given the lack of a comprehensive gene annotation database for Tibetan pigs, we conducted a lift-over of TBPSGs to the human genome to compare the adaptation patterns with hypoxia studies of Tibetan populations. We annotated TBPSGs using multiple databases (GWAs catalog, HPO, MGI, and DisGeNET), and the results showed that TBPSGs are enriched in phosphorylation, angiogenesis, sodium ion transport, and the reproductive process (Supplementary Figures S3–S5; Supplementary Tables S5 and S6. Notably, disease database enrichment analysis indicated that these genes were associated with white blood cell count, lung capacity, and sleepiness (Supplementary Figure S6 and Table S7). In short, these findings collectively suggest that TBPSGs contribute to TBP adaptations across multiple organs and systems, helping them to thrive in high-altitude environments (Supplementary Table S7).

### 2.3. The Newly Identified Top TBPSGs Explain the Adaption of Cardiorespiratory Function and Fat Metabolism

**Figure 3:**
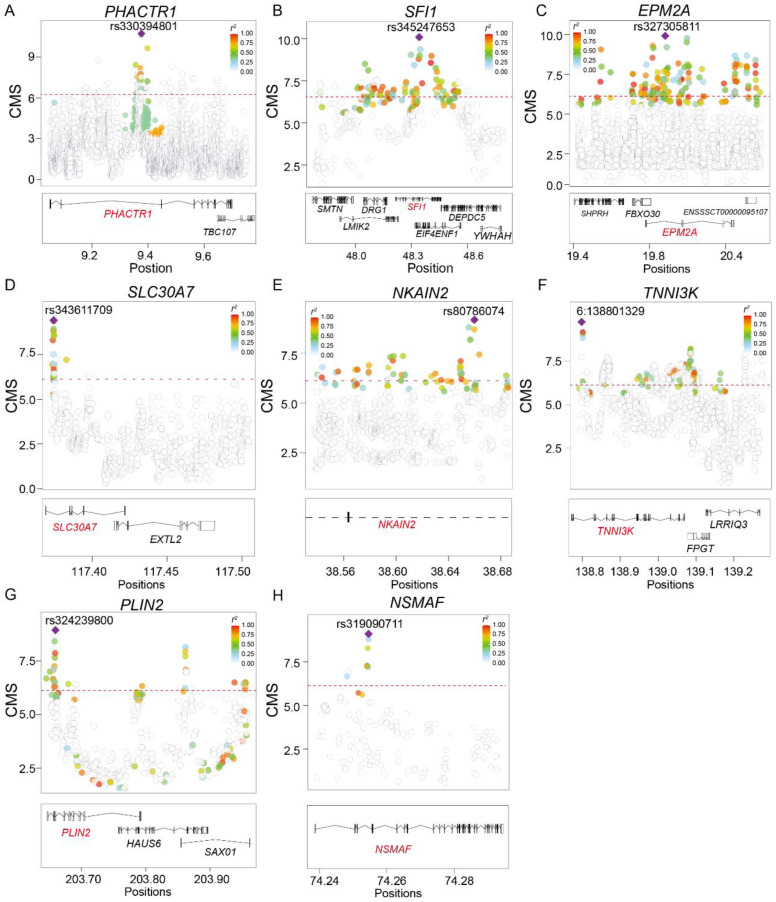
Eight newly identified TBPSGs in the top ten signals, with regional plots of CMS scores shown in panels (**A**–**H**). Peak SNVs are highlighted in color. (**A**) The *PHACTR1* gene region. (**B**) The *SFI1* gene region. (**C**) The *EPM2A* gene region. (**D**) The *SLC30A7* gene region. (**E**) The *NKAIN2* gene region. (**F**) The *TNNI3K* gene region. (**G**) The *PLIN2* gene region. (**H**) The *NSMAF* gene region. Plink2 (https://www.cog-genomics.org/plink/2.0/, accessed on 10 May 2023) was used to calculate recombination rates (r^2^), indicating the degree of linkage disequilibrium (LD) between peak SNVs and other SNVs, with color coding representing different LD levels. The CMS significance threshold is marked by the red dashed line (CMS = 5.66, top 0.05%).

Tibetan pigs are well adapted to the high-altitude low-oxygen environment, characterized by robust cardiopulmonary function and vascular systems. For the top 10 TBPSGs, 8 genes (*PHACTR1*, *SFI1*, *EPM2A*, *SLC30A7*, *NKAIN2*, *TNNI3K*, and *PLIN2*) were newly identified genes subject to strong natural selection (Table 1, Figure 3, and Supplementary Figure S6). Function annotation showed that newly identified TBP-selected genes may be associated with better cardiorespiratory function and fat metabolism function, providing new insights into these adaptive phenotypes. *PHACTR1* (Phosphatase and Actin Regulator 1) belongs to a family of phosphatase and actin regulatory proteins, capable of binding actin and regulating actin cytoskeleton remodeling. *PHACTR1* is enriched with multiple TBPSSs, exhibiting significant frequency and haplotype differences compared to other plain breeds (Figure 3A, Table 1, and Supplementary Table S7). *SFI1*, the peak SNV rs345247653, is highly diverged between Yorkshire and Duroc pigs (PBS = 0.63) and showed significant LD decay (iHS = 5.09 and XPEHH = 3.62) (Figure 3B, Table 1, Supplementary Table S7). Furthermore, we also identified a missense *SFI1* (rs80864866) variant under positive selection (PBS = 0.12, iHS = 3.43, and XPEHH = 3.33) (Supplementary Table S4). *TNNI3K* encodes a protein belonging to the MAPKKK protein kinase family, showing strong positive selection signatures (PBS = 0.39, iHS = 4.89, and XPEHH = 3.59) (Figure 3F, Supplementary Table S7). *EPM2A*, the peak SNV rs327305811, shows strong positive selection in the Tibetan pig, with >43% higher frequency, and a distinctive LD decay pattern compared to other migration pigs (TBP = 0.88, YKS = 0.45, Drouc = 0.43, His = 6.16) (Figure 3C, Table 1, and Supplementary Table S2).

Several TBPSGs are involved in fat metabolism (Figure 3D, Table 1). *SLC30A7*, the peak SNV rs343611709, showed remarkable frequency enrichment and LD decay (PBS = 0.55, XPEHH = 4.39) (Figure 3D, Table 1, Supplementary Table S2). Homozygous knockout mice exhibit low zinc levels, reduced food intake, poor weight gain, and a significant reduction in body fat accumulation, leading to a lean phenotype (Supplementary Table S7). *NSMAF* encodes a WD repeat protein essential for TNF-mediated neutral sphingomyelinase activation, potentially regulating TNF-induced cellular responses such as inflammation, consistent with the sphingolipid metabolism-related processes observed in our GO enrichment analysis (Figure 2D and Figure 3H). The peak SNV rs324239800 is located in the intronic region of *PLIN2* and shows a strong positive selection in Tibetan pigs, with > 71% higher frequency, and a distinctive LD decay pattern compared to other migration pigs (TBP = 0.72, YKS = 0.02, Duroc = 0.03) (Figure 3G, Table 1, and Supplementary Table S2). 

## 3. Discussion

The adaptation of Tibetan pigs to high altitudes is a multi-organ and multi-system process, exhibiting many typical adaptive traits which likely have a genetic basis. In this study, we used well-controlled whole-genome data (GWS) and the composite of multiple signals method to systematically identify the Darwinian positive selection signals across the Tibetan pig whole genome. Our experiments yielded a confident set of 32,499 TBPSSs and 241 TBPSGs, with 213 genes being newly reported genes in this study, providing new insights into the genetic mechanisms underlying the adaptive phenotypes of Tibetan pigs.

Tibetan pigs, a typical high-altitude-adapted breed in China [11], harbor adaptive mutations through long-term natural selection. High-altitude-adapted animals and humans are classic examples for hypoxia adaptation evolutionary study. Numerous studies have identified *EPAS1* and *EGLN1* as key genes for high-altitude adaptation, and structural variants (SVs) play a role in *EPAS1* function [23,24,25]. We employed the CMS method [8] and overcame methodological biases to identify 32,449 adaptive SNVs and 241 positive selected genes. Here, we selected only the top 0.05% of genome-wide SNVs, leading to the identification of a robust set of natural selection genes. Notably, we employed two control groups, each with a sample size greater than 25, thereby effectively eliminating the statistical errors in mutation rate estimates due to small sample sizes, as well as the confounding effects of population structure and ancestral admixture. This ensures the reliability of genes under natural selection. Additionally, we chose the genes with the strongest positive selection signal as the peak genes for each independent region, possibly excluding genes in strong linkage disequilibrium with the peak gene, such as the *SFI1* gene, as *DRG1* is strongly linked to *SFI1*, with an r^2^ > 0.6 (Figure 3B). 

Tibetan pig adaptation to high altitudes involves a multi-system process, characterized by gene pleiotropy or multi-gene effects at the genetic level. Overall, the 241 genes we identified functionally support the notion that the adaptation of Tibetan pigs to high altitudes is a multi-organ and multi-system process. Among the 241 TBPSGs, 213 are newly identified genes. Of the top ten genes, eight TBPSGs are newly identified, with four genes (*PHACTR1*, *SFI1*, *TNNI3K*, and *EPM2A*) being highly expressed in the cardiopulmonary system and thought to play key roles in cardiopulmonary function regulation [26,27]. A large-scale human population GWA study showed that multi-SNVs in *PHACTR1* are significantly associated with myocardial infarction, coronary artery disease, and arterial function [26]. *SFI1*, a centriolar protein, regulates centriole replication, with polymorphisms linked to myocardial infarction, coronary artery disease, and carotid artery dissection susceptibility (HPO) [28]. *TNNI3K* plays a key role in cardiac physiology by inhibiting cardiomyocyte division and is associated with cardiac and glucose metabolism [27,29]. Studies suggest that *TNNI3K* may regulate atrial myocardial cell function in pigs [30]. Homozygous knockout mice show a reduced response to ischemia/reperfusion injury, suggesting a protective role against heart disease [31]. *EPM2A* encodes a dual-specificity phosphatase involved in carbohydrate metabolism and glycogen stability maintenance [32]. Mouse knockout models exhibit significant phenotypic alterations across several biological processes, including the cardiovascular system, growth and development, muscle function, and metabolic stability. During the hypoxia adaptation, improvements in cardiopulmonary function have been observed in Tibetan pigs [8], yaks [27], Tibetan sheep [25], and plateau zokors [33], suggesting that these genes may contribute to enhanced cardiopulmonary function in Tibetan pigs.

Notably, genes such as *PHACTR1*, *EPM2A*, and *PLIN2* show strong positive selection in Tibetan pigs, being significantly associated with both cardiopulmonary function and reproductive and ocular development [1,5,18,28]. This suggests that these genes contribute to the fitness maintenance and adaptation to strong ultraviolet radiation in Tibetan pigs. 

Additionally, cold is a typical condition of high altitudes, and effective fat metabolism is crucial for maintaining body temperature and surviving harsh conditions. Our study not only identified multi-genes related to fat metabolism among the top 10 TBPSGs but also found that the *KHDRBS2* gene, previously reported in Tibetans, is associated with cardiopulmonary function and lipid metabolism [8]. Several TBPSGs are involved in fat metabolism. *SLC30A7*, a member of the zinc transporter (ZNT)/SLC30 subfamily, functions to regulate oxidative stress. *NSMAF* is also implicated in carcass weight, bone density, and growth in Red Angus cattle [34]. *NKAIN2* encodes a transmembrane protein interacting with the β subunit of sodium/potassium-transporting ATPase. Studies show significant associations with backfat thickness and enrichment in adipogenesis and energy homeostasis pathways [35]. Furthermore, large-scale population GWASs have demonstrated a significant association between *NKAIN2* and hemoglobin concentration (HGB) [36]. *PLIN2* is associated with lipid droplet membrane material, potentially being involved in adipose tissue development and maintenance [37]. Large-population GWASs showed that *PLIN2* was associated with red blood cell distribution width (Supplementary Table S7). Hence, the Tibetan pig’s enriched allele of *PLIN2* may improve oxygen transport and fat metabolism. High-altitude hypoxia poses significant challenges to energy metabolism, affecting the body systemically. Therefore, Tibetan pigs likely selected a set of genes to improve energy metabolism regulation in response to hypoxia. 

Collectively, we systematically analyzed whole-genome sequencing data of Tibetan pigs and identified a confident set of 240 genes (TBPSGs) exhibiting strong signals of positive natural selection. This finding underscores the role of gene pleiotropy in the adaptation of Tibetan pigs to high-altitude environments, suggesting that these genes may collectively contribute to shaping their adaptive traits. The functions of the eight newly identified TBPSGs may synergistically enhance cardiopulmonary function and fat metabolism under hypobaric hypoxia. Future functional studies are needed to elucidate the underlying regulatory mechanisms and resultant phenotypic outcomes. Our study provides genetic insights into high-altitude adaptive evolution and the specific adaptive phenotypes of Tibetan pigs.

## 4. Materials and Methods

### 4.1. Samples and Sequencing

We collected 140 WGS data from distinct pig breeds, with 30 samples generated in this study and 110 samples collected from previously published research (Supplementary Table S1). A total of 30 unrelated samples were collected, including 10 Tibetan pigs from Hezuo, Gansu (altitude 3000 m). To ensure the breed purity of Yorkshires and Durocs, we obtained 20 purebred pig samples (10 Yorkshire pigs and 10 Duroc pigs) from the breeding base in Gaotai, Gansu (altitude 800 m), and whole-genome sequencing was performed on the Illumina NovaSeq platform, generating an average of 47 Gb of data per individual and a mean depth of 12.13×. The average Q30 score was 92.13.

### 4.2. Quality Control and Alignment

Raw reads underwent quality control using FastQC (https://www.bioinformatics.babraham.ac.uk/projects/fastqc/, v 0.12.0, accessed on 3 May 2023), and low-quality reads were removed. High-quality reads were aligned to the pig reference genome (genome assembly: Sscrofa11.1 (GCA_000003025.6) using BWA-MEM. PCR duplicate reads were marked using the GATK4 MarkDuplicates function, and the reads were sorted with SAMtools.

### 4.3. Variant Calling

Using the Genome Analysis Toolkit (GATK), all 140 samples were subjected to realignment, recalibration, and variant analysis. The analysis followed the good practice workflows recommended by GATK4. Variants were identified for each sample using the HaplotypeCaller module, followed by joint genotyping and hard filtering to balance accuracy and sensitivity in the final multi-sample call set. Finally, a hard filter was applied using the following GATK filter expression: “QD < 2.0 || MQ < 40 || FS > 60.0 || SOR > 3.0 || MQRankSum < −12.5 || ReadPosRankSum < −8.0”. This process identified 27,614,561 variants across the 140 pig samples.

### 4.4. Data Quality Control and Variant Annotations

#### 4.4.1. Quality Control at Sample Level

For the 140 samples, strict quality control (QC) steps were performed before subsequent analyses. We excluded 9 individuals with high missing rates (5%) or abnormal heterozygosity. Individuals with a genotype failure rate ≥5% and heterozygosity deviating ±3 standard deviations from the mean were also excluded (8). Additionally, 2 duplicates and 11 admixed lineage samples were removed. Ultimately, 118 samples passed individual-level QC. Principal component analysis (PCA) was conducted using EIGENSOFT (https://reich.hms.harvard.edu/software, v 7.2.1, accessed on 3 May 2023), considering linkage disequilibrium (LD) effects, and the data were pruned using PLINK2.0.

#### 4.4.2. Quality Control at SNVs Level

For the 118 samples that passed individual-level QC, VCFtools was used to classify the mutations into 22,386,319 single-nucleotide variants (SNVs) and 5,228,242 indels. The following steps were then applied:

Removal of singletons;

Removal of SNVs with missing genotype data exceeding 5%;

Removal of SNVs with significant deviation in the Hardy–Weinberg equilibrium test (*p* < 1 × 10^−6^).

#### 4.4.3. Variant Annotations

After these QC steps, we finalized a dataset containing 13,008,482 SNVs. After two quality control steps, we calculated allele frequencies and retained only biallelic SNVs for downstream analysis. A total of 13,002,441 biallelic SNVs were identified and functionally annotated using ANNOVAR (https://annovar.openbioinformatics.org; SnpEff, http://pcingola.github.io/SnpEff/; VEP https://www.ensembl.org/info/docs/tools/vep/, accessed on 12 May 2023).

### 4.5. Detection of Genomic Signatures of Positive Selection

To detect genome-wide signatures of positive selection in Tibetan pigs, we used haplotype-based methods (XPEHH and iHS) and allele frequency-based methods (*F*_ST_ and Tajima’s D test). The composite of multiple signals (CMS) score for each variant was estimated by combining iHS, XPEHH, DAF, and PBS statistics, resulting in 12,998,482 SNVs used for detecting positive selection.

#### 4.5.1. Population Structure Analysis

For the merged dataset, after quality control (QC) and LD pruning, we ran ADMIXTURE (v1.3.0) five times with random seeds for each K value from 2 to 5. ADMIXTURE cross-validation error estimates were generated to determine the optimal K value.

#### 4.5.2. *F*_ST_

Allele frequencies and pairwise genetic distances (*F*_ST_) of the whole-genome SNVs were calculated using PLINK (https://www.cog-genomics.org/plink2, v.2, accessed on 12 May 2023) with the commands (*--freq*, *--fst*).

#### 4.5.3. Linkage Disequilibrium (LD) Decay

LD decay for TBP, YKS, and Duroc was evaluated using PopLDdecay software (https://github.com/BGI-shenzhen/PopLDdecay, v 3.4.3, accessed on 12 May 2023) with default parameters.

#### 4.5.4. Population Branch Statistic (PBS)

Principal component analysis (PCA) and admixture results indicated that our control samples are representative (Figure 1, Supplementary Figure S1). By integrating these results with previously published data, we obtained 60 control samples (25 Duroc pigs and 35 Yorkshire pigs). The numbers of individuals in each group exceeded 20, ensuring the reliability of allele frequency estimates. Moreover, the two control groups effectively minimized the impact of population structure and potential ancestral confounding factors, thereby enhancing the reliability of detecting genes under natural selection. The PBS scores for Tibetan pigs (TBPs) were calculated using the following formula:

PBS_TBP_ = (*F*_ST(TBP-YKS)_ + *F*_ST(TBP-Duroc)_ − *F*_ST(YKS-Duroc)_)/2

#### 4.5.5. XPEHH and iHS

Selscan [38] was used to calculate XPEHH and iHS. Whole-genome normalization was performed, excluding SNVs where EHH decay was below 0.05.

#### 4.5.6. CMS

The CMS scores for genome-wide SNVs of Tibetan pigs were calculated by combining iHS, XPEHH, DAF, and PBS statistics, following the formula from a previous study [39]. We identified the top 0.5% of SNVs with the highest CMS scores (32,499 SNVs) as candidate sites under positive selection in Tibetan pigs. We further filtered these to retain only those SNVs significantly enriched in Tibetan pig populations. Finally, we performed LD-based clustering to identify independent signal regions (r^2^ ≤ 0.2, cluster window size 500 kb). We identified 270 independent positive selection regions involving 241 genes, with 29 regions not overlapping any gene. In each independent region (1 Mb block), the gene with the strongest selection signal was designated as a Tibetan pig positively selected gene (TBPSG), with the peak SNV located within 5 kb upstream or downstream of the gene body. 

#### 4.5.7. Enrichment Analysis

Functional enrichment analysis, including pathway (KEGG) and biological process (GO) annotations of TBPSGs, was performed using g:Profiler (https://biit.cs.ut.ee/gprofiler/gost, accessed on 12 May 2023) and Metascape [40].

## Figures and Tables

**Table 1:** The top 10 TBPSGs in Tibetan pigs.

Chromosome	rsID	Position (*Sus scrofa 11.2*)	CMS	*PBS*	Genes	Enriched Allele Frequency		
						TBPs (*n* = 58)	DU (*n* = 25)	YKS (*n* = 35)
7	rs330394801	9366281	11.14	0.63	*PHACTR1*	0.93	0.33	0.06
14	rs345247653	48268185	10.45	0.53	*SFI1*	0.92	0.27	0.3
1	rs327305811	19895804	9.92	0.43	*EPM2A*	0.88	0.45	0.43
4	rs345409819	49294816	9.72	0.59	*BCR*	0.93	0.25	0.36
4	rs343611709	117365692	9.42	0.55	*SLC30A7*	0.77	0.17	0.12
1	rs80786074	38655025	9.21	0.59	*NKAIN2*	0.81	0.08	0.2
6	6:138801329	138801329	9.17	0.38	*TNNI3K*	0.76	0.01	0.31
1	rs344915163	203690850	9.11	0.67	*PLIN2*	0.75	0.02	0.03
18	rs333217490	40016420	9.09	0.34	*BBS9*	0.68	0.25	0.01

Note: rsID, rs number in dbp-138; Position, the physical distance in the *Sus scrofa 11.2*; DU, Duroc; YKS, Yorkshire. TBPs, Tibetan pigs. Red and black represent newly identified and reported genes.

## Data Availability

The data generated in this study are available through the Genome Sequence Archive (GSA: https://ngdc.cncb.ac.cn/, accessed on 6 October 2024) for VCF format, with BioProject accession number PRJCA031364.

## Supplementary Materials

The following supporting information can be downloaded at: https://www.mdpi.com/article/10.3390/ijms252011303/s1.
