# Supplementary material for: Genetic Adaptations of the Tibetan Pig to High-Altitude Hypoxia on the Qinghai–Tibet Plateau
Source: Int J Mol Sci. 2024 Oct 21;25(20):11303. doi: 10.3390/ijms252011303 (PMC11508817; doi:10.3390/ijms252011303)
Supplement: Supplementary file 1 [file ijms-25-11303-s001.zip › TBP.S.10.17(1).pdf]

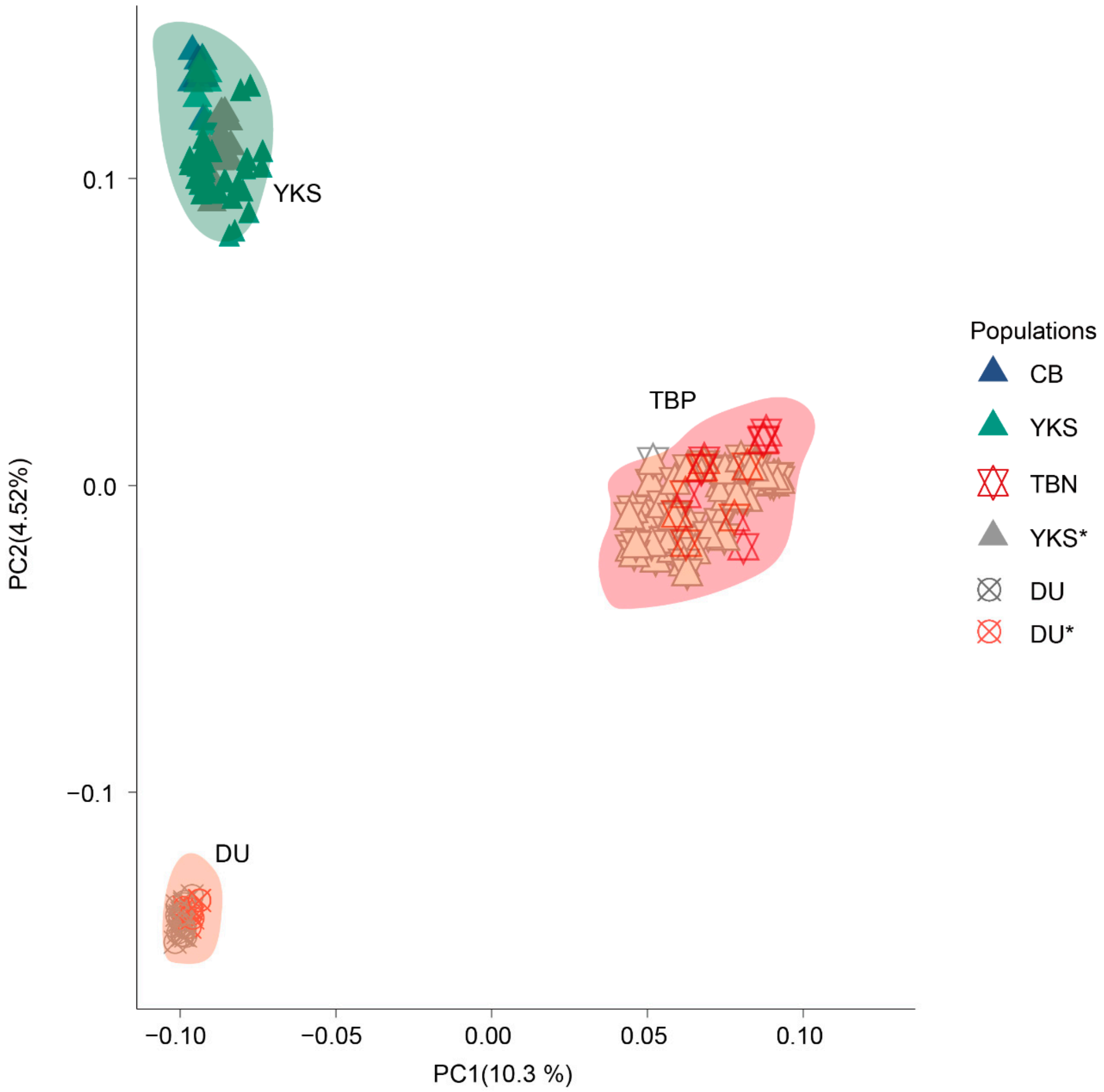

Supplementary Figure S1 The 30 newly generated whole-genome sequences (WGS) are consistent with the 110 previously published WGS datasets, indicating good representativeness of our samples. Principal component analysis (PCA) revealed significant separation of Tibetan pigs, YKS, and Duroc along PC1, confirming that our newly generated individuals do not differ in ancestral components from the published individuals.

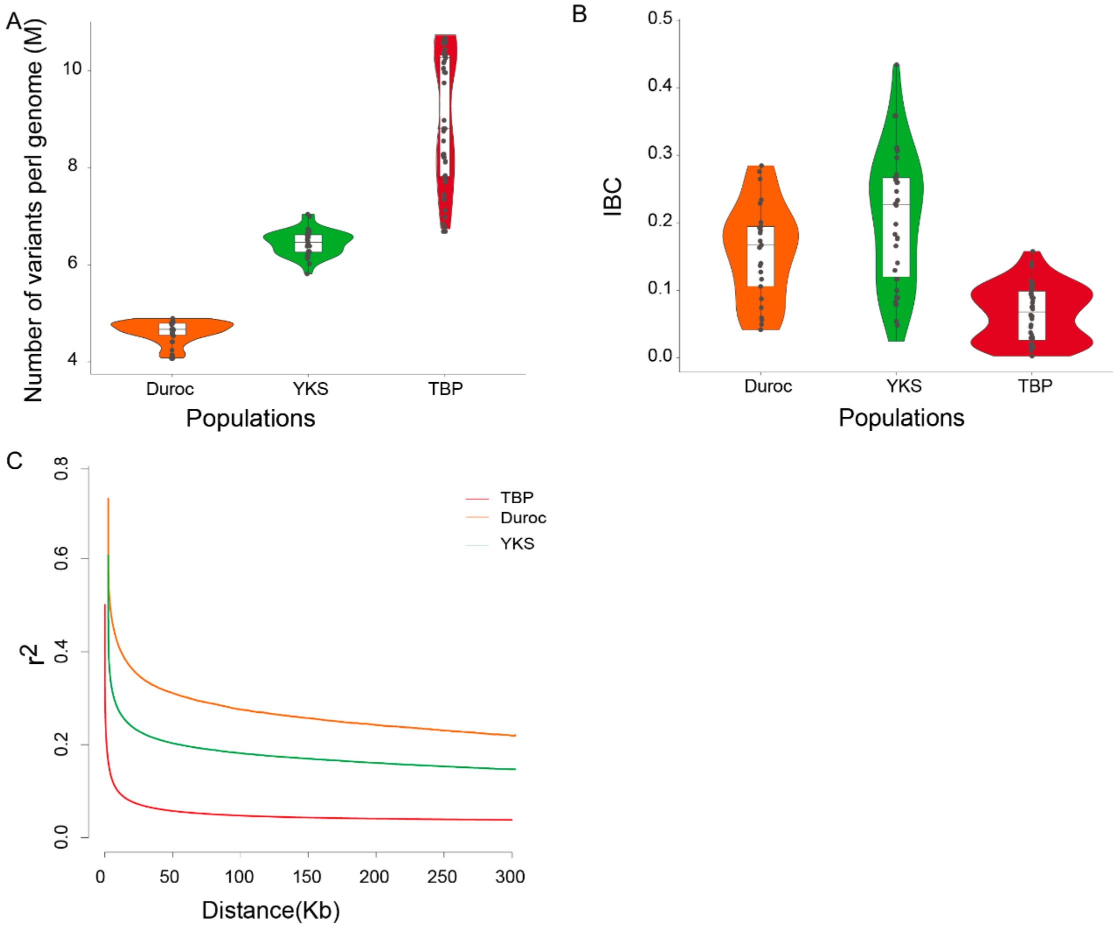

Supplementary Figure S2 Tibetan pigs exhibit the highest genetic diversity. A. the number of mutations carried by individuals and the inbreeding coefficient are key indicators of genetic diversity. On average, Tibetan pigs carry 8.99 million mutations, significantly higher than YKS (p = 2.82e-17) and Duroc (p = 1.72e-27). B. the inbreeding coefficient (IBC) represents the increase in allele homozygosity in the genome due to inbreeding, which reflects genetic diversity to some extent. Tibetan pigs have the lowest inbreeding coefficient, indicating their high genetic diversity. C. linkage disequilibrium (LD) decay indicates genetic structure and is associated with genetic diversity. Tibetan pigs exhibit the slowest LD decay, supporting their high genetic diversity.

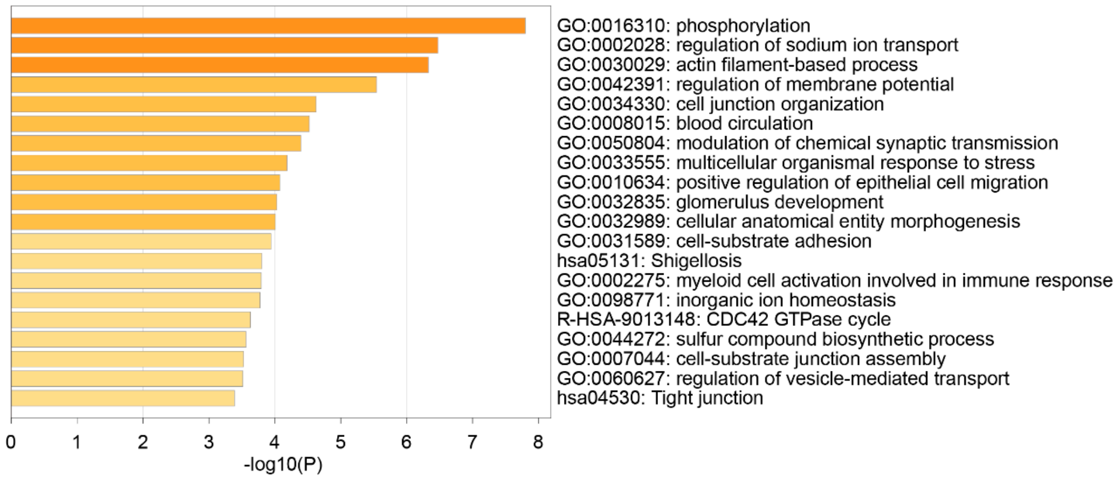

Supplementary Figure S3 GO Enrichment Analysis of 241 TBPSGs. We mapped the 241 Tibetan Pig Positively Selected Genes (TBPSGs) to the human genome and performed Gene Ontology (GO) enrichment analysis using a human regulatory database. The results corroborated the GO enrichment analysis based on the pig database, consistently highlighting enrichment in the phosphorylation term.

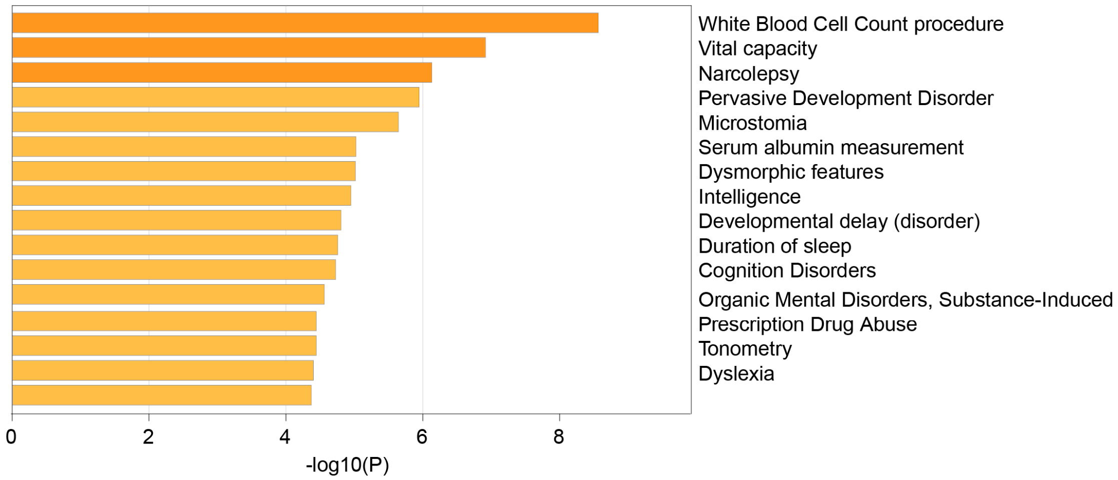

Supplementary Figure S4 DisGeNET Enrichment Analysis of 241 TBPSGs. We conducted an enrichment analysis of the 241 Tibetan Pig Positively Selected Genes (TBPSGs) using the DisGeNET database. The analysis revealed that these genes are associated with traits such as white blood cell count, lung capacity, and sleepiness.

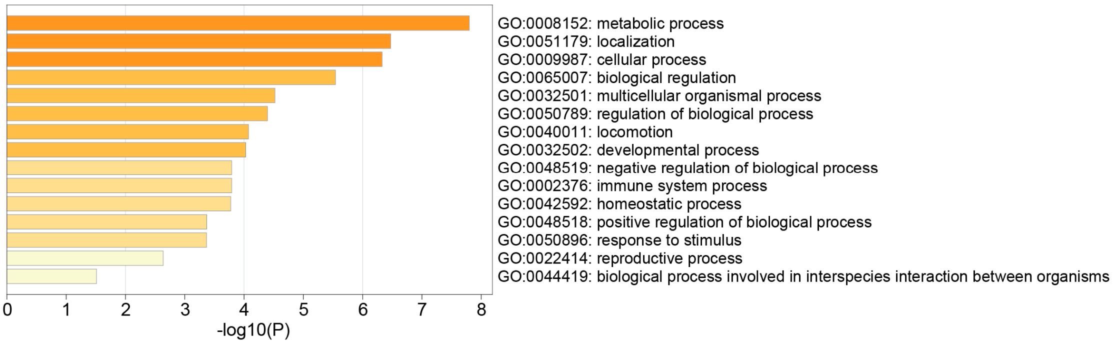

Supplementary Figure S5 Biological Process Enrichment of 241 TBPSGs. By broadening our focus to encompass higher-level biological processes, we observed that the 241 Tibetan Pig Positively Selected Genes (TBPSGs) are significantly enriched in pathways related to metabolism, immune response, and reproduction.

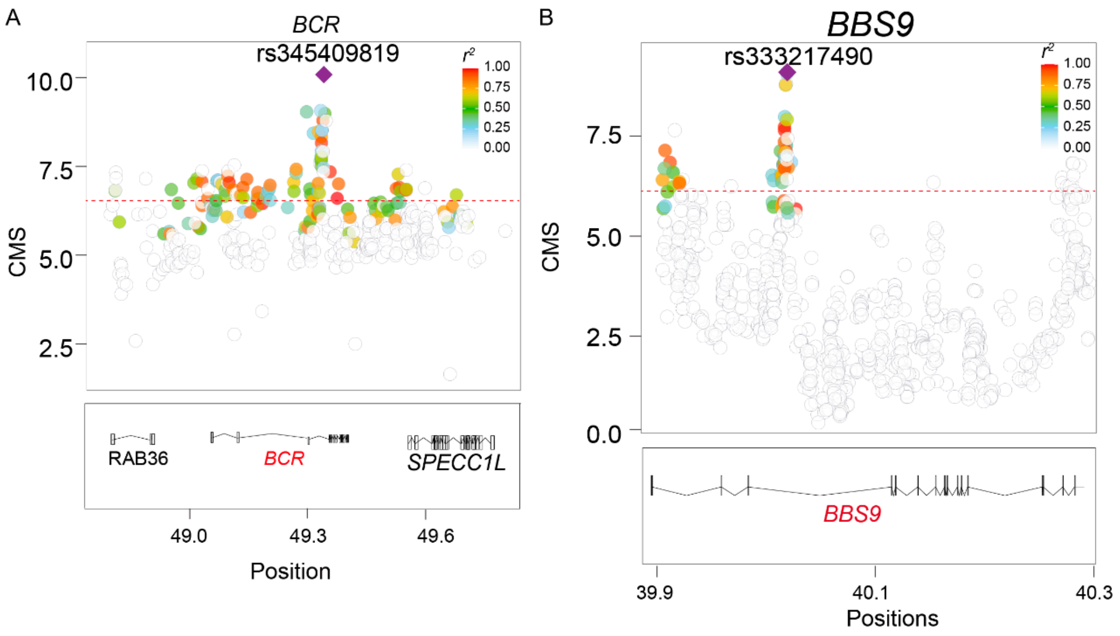

Supplementary Figure S6 Two reported TBPSGs in the top 10 signals, with regional plots of CMS scores shown in panels A–B. Peak SNVs are highlighted in color. A. *BCR* gene region. B. *BBS9* gene region.
